# Identification of a nuclear localization motif in the serine/arginine protein kinase PSRPK of *physarum polycephalum*

**DOI:** 10.1186/1471-2091-10-22

**Published:** 2009-08-25

**Authors:** Shide Liu, Zhuolong Zhou, Ziyang Lin, Qiuling Ouyang, Jianhua Zhang, Shengli Tian, Miao Xing

**Affiliations:** 1 Shenzhen Key Laboratory of Microbial Genetic Engineering and College of Life Science, Shenzhen University, Shenzhen, PR China, 518060; 2 Institute of Photoelectron, Shenzhen University, Shenzhen, PR China, 518060

## Abstract

**Background:**

Serine/arginine (SR) protein-specific kinases (SRPKs) are conserved in a wide range of organisms, from humans to yeast. Studies showed that SRPKs can regulate the nuclear import of SR proteins in cytoplasm, and regulate the sub-localization of SR proteins in the nucleus. But no nuclear localization signal (NLS) of SRPKs was found. We isolated an SRPK-like protein PSRPK (GenBank accession No. DQ140379) from *Physarum polycephalum *previously, and identified a NLS of PSRPK in this study.

**Results:**

We carried out a thorough molecular dissection of the different domains of the PSRPK protein involved in its nuclear localization. By truncation of PSRPK protein, deletion of and single amino acid substitution in a putative NLS and transfection of mammalian cells, we observed the distribution of PSRPK fluorescent fusion protein in mammalian cells using confocal microscopy and found that the protein was mainly accumulated in the nucleus; this indicated that the motif contained a nuclear localization signal (NLS). Further investigation with truncated PSPRK peptides showed that the NLS (^318^PKKGDKYDKTD^328^) was localized in the alkaline Ω-loop of a helix-loop-helix motif (HLHM) of the C-terminal conserved domain. If the ^318^PKKGDK^322 ^sequence was deleted from the loop or K^320 ^was mutated to T^320^, the PSRPK fluorescent fusion protein could not enter and accumulate in the nucleus.

**Conclusion:**

This study demonstrated that the ^318^PKKGDKYDKTD^328 ^peptides localized in the C-terminal conserved domain of PSRPK with the Ω-loop structure could play a crucial role in the NLS function of PSRPK.

## Background

Serine/arginine (SR) protein-specific kinases (SRPKs) represent a class of evolutionarily conserved kinases that specifically phosphorylate the arginine/serine-rich (RS) domains of the SR splicing factor [1]. After the identification of SRPK1–the first SRPK–by Gui *et al*. [2,3], other SRPKs such as SRPK2 [4], mouse SRPK1 and SRPK2 [5], yeast Dsk1 [6] and Sky1p [7], nematode SPK-1 [8], *Trypanosoma cruzi *TcSRPK [9], and *Arabidopsis thaliana *SRPK4 [10] have been subsequently identified. Some studies have shown that SRPKs are mainly localized in the cytoplasm, with only a few present in the nucleus [[2,5], and [11]]. The SRPKs that are localized in the cytoplasm regulate the nuclear import of SR proteins via phosphorylation [12], while those in the nucleus regulate the nuclear localization of the SR splicing proteins via phosphorylation [13-15]. Ding *et al*. [11] have discovered that the spacer sequences present between the conserved domains of mammalian SRPKs have cytoplasmic anchoring function. However, there has been no report on the nuclear localization signal (NLS) of SRPKs.

Model organism *Physarum polycephalum *is mitochondria-containing primitive eukaryotes. Its life cycle includes a single-celled amoeba, plasmodium (the main life form), and sporulation stages. The nuclei in the same plasmodium proliferate by way of synchronization of mitosis. In our previous study, we identified an SRPK containing 426 amino acids (aa) from *P. polycephalum*; this kinase was termed PSRPK (GenBank accession No. DQ140379). Similar to other SRPKs, PSRPK also has 2 conserved domains and can phosphorylate human SR protein alternate splicing factor/splicing factor 2 (ASF/SF2). However, it differs from other SRPKs in that the divergent motif (≥ N) in its N-terminus is rich in acidic amino acids; the spacer sequence of PSRPK between two conserved domains is shorter than that of other SRPKs. In this study, the distribution of PSRPK fluorescent fusion protein in mammalian cells was observed using laser scanning confocal microscopy. Maximum fluorescence was detected in the nucleus, suggesting the existence of an NLS in PSRPK. When the distribution of truncated PSPRK peptides was observed, a putative NLS was found in the C-terminal of PSRPK based on its homology to the classic NLS. When the sequence was deleted or when lysine 320 in the sequence was substitute by threonine, the cytoplasmic distribution of PSRPK was observed.

## Methods

### Construction of expression plasmids containing PSRPK and truncated PSPRK peptides

pMD18-psrpk had been constructed previously [16]. PSRPK fragments were obtained by polymerase chain reaction (PCR), using pMD18-psrpk as the template and PSRPK-specific primers (Table 1). The resulting PCR fragments and the mammalian expression vectors pDsRed1-N1 and pECFP-C1 (Clontech) were digested with *Eco*RI and *Bam*HI. The digested fragments were subjected to agarose gel electrophoresis, recovered from the gel, purified, and ligated using T4 DNA ligase; this resulted in the expression plasmids pDsRed-*psrpk *and pECFP-*psrpk*.

**Table 1:** Primers used for cloning *psrpk *and *tps*

	DNA fragmants	sense and anti-sense primers
*psrpk*	1~1278	F1:5'-CCGGAATTCTATGGAAAACATATTCAAGGAGAAGG-3' R1: 5'-ACGCGTCGACAGAAATGGAGGCACATCAGCC-3'
*tp1*	84~1278	5'-CCGGAATTCTATGGATAGCGAAGATGAGGGAAC-3'/R1
*tp2*	1~588	F1/5'-ACGCGTCGACAGCACGTTTTCAGGTTTGAG-3'
*tp3*	589~1278	5'-CCGGAATTCTATGGACCATCTGTTACGACCAG-3'/R1
*tp4*	778~1278	5'-CCGGAATTCTATGAAAATCGCCGATCTAGGCAC-3'/R1
*tp5*	589~951	F2: 5'-CCGGAATTCATGGACCATCTGTTACGACCAGACAC-3' R2: 5'-ACGCGTCGACTGGCAAAAAAGGAGATCTCCGGTGG-3'
*tp6*	589~1029	F2/5'-ACGCGTCGACTGGCGCGGCATTCTTCCTAGTAGCTC-3'
*tp7*	985~1278	F3: 5'-ACGCGTCGACGATGGATCACTTGGCTTTGATGATTG-3' R3: 5'-ATGCGGATCCGCCAGAAATGGAGGCACATCAGCC-3'
*tp8*	589~951 985~1278	F2/R2 F3/R3
*tp9*	1~951 985~1278	F4: 5'-CCGGAATTCATGGAAAACATATTCAAGGAGAAG-3'/R2 F3/R3

**Figure 1:**
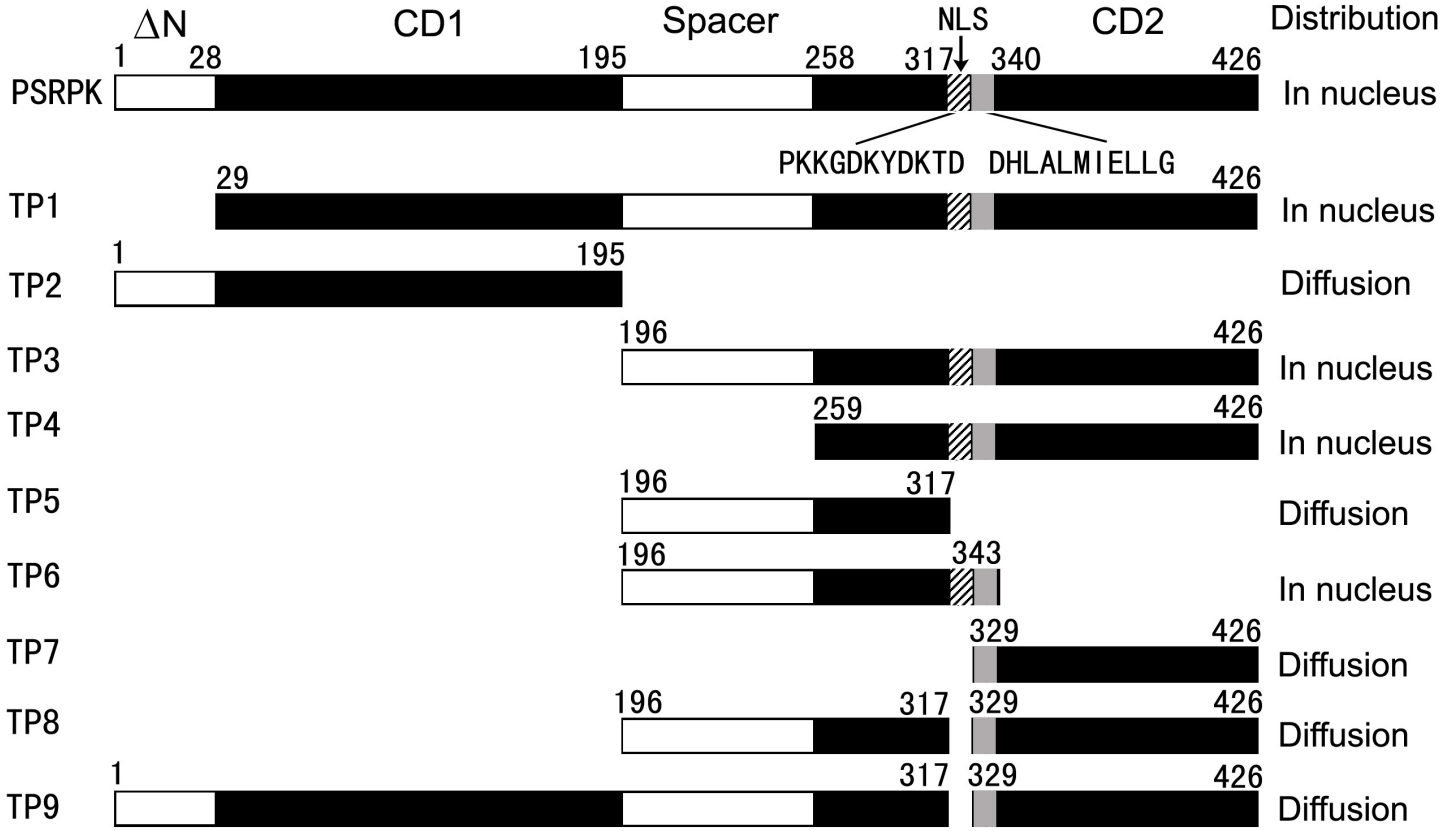
**Distribution of fluorescent fused PSRPK in mammalian cells**. a and b: DAPI staining nuclear of cells HeLa and L929 containing pECFP-*psrpk *were observed under a BX51 fluorescent microscope (Olympus, 400×); c and d: distribution of CFP-PSRPK in cells HeLa and L929 were observed under a confocal microscope (TCS SP2, Leica).

We designed the following truncated PSRPK peptides (Figure 1): TP1 (≥ N deletion), TP2 (spacer sequence and CD2 deletions), TP3 (≥ N and CD1 deletions), TP4 (≥ N, CD1, and spacer sequence deletions), TP5 (without the ^318^PKKGDKYDK^326 ^and ^329^DHLALMIELLG^339 ^sequences), TP6 (containing ^318^PKKGDKYDK^326 ^and ^329^DHLALMIELLG^339 ^sequences), TP7~TP9 (all without the ^318^PKKGDKYDKTD^328 ^sequence; further, TP7, 329~426 aa; TP8, 197~317 aa combined with 329~426 aa; TP9, 1~317 aa combined with 329~426 aa). Recombinant plasmids were constructed using the primer pairs (Table 1) and the pMD18-*psrpk*, pDsRed1-N1, and pECFP-C1 vectors, resulting in pECFP-*tp1*~pECFP-*tp4*, pDsRed-*tp5*, pDsRed-*tp6*, pDsRed-*tp7*, pDsRed-*tp8*, and pDsRed-*tp9*.

### Construction of expression plasmids containing mutant and default PSRPK peptides

In order to understand the effect of the loop motif on nuclear localization, we designed default PSRPK, namely, PSRPK^d ^(^318^PKKGDK^323 ^deletion) and mutant PSRPK, namely, PSRPK^m ^(K^320 ^→ T^320 ^mutation). The DNA fragments containing *psrpk*^*d *^or *psrpk*^*m *^up- and downstream were obtained using the primers listed in Table 2 and pMD18-*psrpk *as the template. The 2 overlapping PCR products were mixed in a ratio of 1:1, followed by denaturing at 94°C for 3 min and annealing at 55°C for 3 min. Since there were 18 complementary bases (underlined parts of primers, Table 2) in the 2 PCR fragments, they would be complementary to the cohesive ends during annealing at 55°C. The DNA with 5' and 3' overhangs could be extended by DNA polymerase (Hot Start Taq Polymerase; Qiagen) to form a complete DNA fragment at 72°C. Primer pair F4/R3 were introduced in the PCR reaction for the amplification of the *psrpk*^*d *^and *psrpk*^*m *^sequences. The 2 PCR products, *psrpk*^*d *^and *psrpk*^*m*^, were inserted into pDsRed1-N1, resulting in the recombinant plasmids pDsRed-*psrpk*^*d *^and pDsRed-*psrpk*^*m*^, respectively.

**Table 2:** Primers used for cloning *psrpk*^*d *^and *psrpk*^*m*^

	DNA fragmants	Sense and anti-sense primers
*psrpk*^*d*^	1~951 969~1278	F4/5'-GCAAAAAAGGAGATCTCCGGTGGCC-3' 5'-GGAGATCTCCTTTTTTGCTATGATAAGACAGAT-3'/R1
*psrpk*^*m*^	959^A ^→ 959^C^	F4/5'-GCAAAAAAGGAGATCTCCGGTGGCC-3' 5'-GGAGATCTCCTTTTTTGCCCCAAAA**C**AGGAG-3'/R3

### Transfection of mammalian cells with lipofectamine

The abovementioned recombinant plasmids were used to transform *E. coli *DH5a cells. The positive recombinant products were grown in Luria-Bertani (LB) medium containing 30 μg/ml kanamycin. The plasmids were isolated using the alkaline lysis method, dissolved in Tris-EDTA (TE) buffer, and quantified using GeneQuant pro (Amersham Bioscience, GE Healthcare). The purified plasmid (A_260_/A_280 _> 1.8) was diluted to 16 μg/ml in serum-free RPMI-1640 medium (Gibco). Lipofectamine™ 2000 (Invitrogen) was diluted in serum-free RPMI-1640 medium (40 μl/ml) and then mixed with an identical volume of plasmid solution, resulting in lipid-DNA complexes.

HeLa and L929 cells (ATCC) were cultured in RPMI-1640 medium supplemented with 10% fetal bovine serum (FBS; Hyclone), 100 IU/ml penicillin, and 100 μg/ml streptomycin at 37°C in a 5% CO_2 _incubator. The cells were harvested while they were in the logarithmic phase and 1.5 ml culture (approximately 1 × 10^5 ^cells) was seeded into a 35-mm plate with a coverslip and cultured for 24 h. The medium was replaced with serum-free RPMI-1640 medium, and the cells were cultured for 1 h to initiate transfection. The cells were overlaid with 200 μl lipid-DNA complexes and cultured for 5 h. The medium was replaced with 1.5 ml of 10% FBS medium, and the cells were further cultured for 48 h.

### Staining of cells with 4',6-diamidino-2-phenylindole (DAPI)

The HeLa cells were transfected with pECFP-*psrpk*, and L929 cells were transfected with pECFP-*psrpk*, pDsRed-*psrpk*, pDsRed-*tp5*~pDsRed-*tp5*, pDsRed-*psrpk*^*d *^and pDsRed-*psrpk*^*m *^as described above, respectively. The transfected cells were incubated on a coverslip for 48 h, following which DAPI staining (Bitian) was performed for 5 min. The cells were washed 3 times (each wash, 5 min) with D-Hands solution to remove DAPI. Fluorescence was visualized under a fluorescence microscope (Olympus BX51, 400×) or a confocal microscope (Leica TCS SP2, 1000×) at 465~495 nm (green) or 560~600 nm (red) in order to observe the distribution of the fluorescent fusion protein in the cells.

### Three-Dimensional Structure Modeling of PSRPK

The three-dimensional (3-D) PSRPK structure was modeled by the Swiss Institute of Bioinformatics program SWISS-MODEL, available at , and the results analyzed by the DeepView program [17].

## Results

### PSRPK was mainly localized in the nucleus of mammalian cells

**Figure 2:**
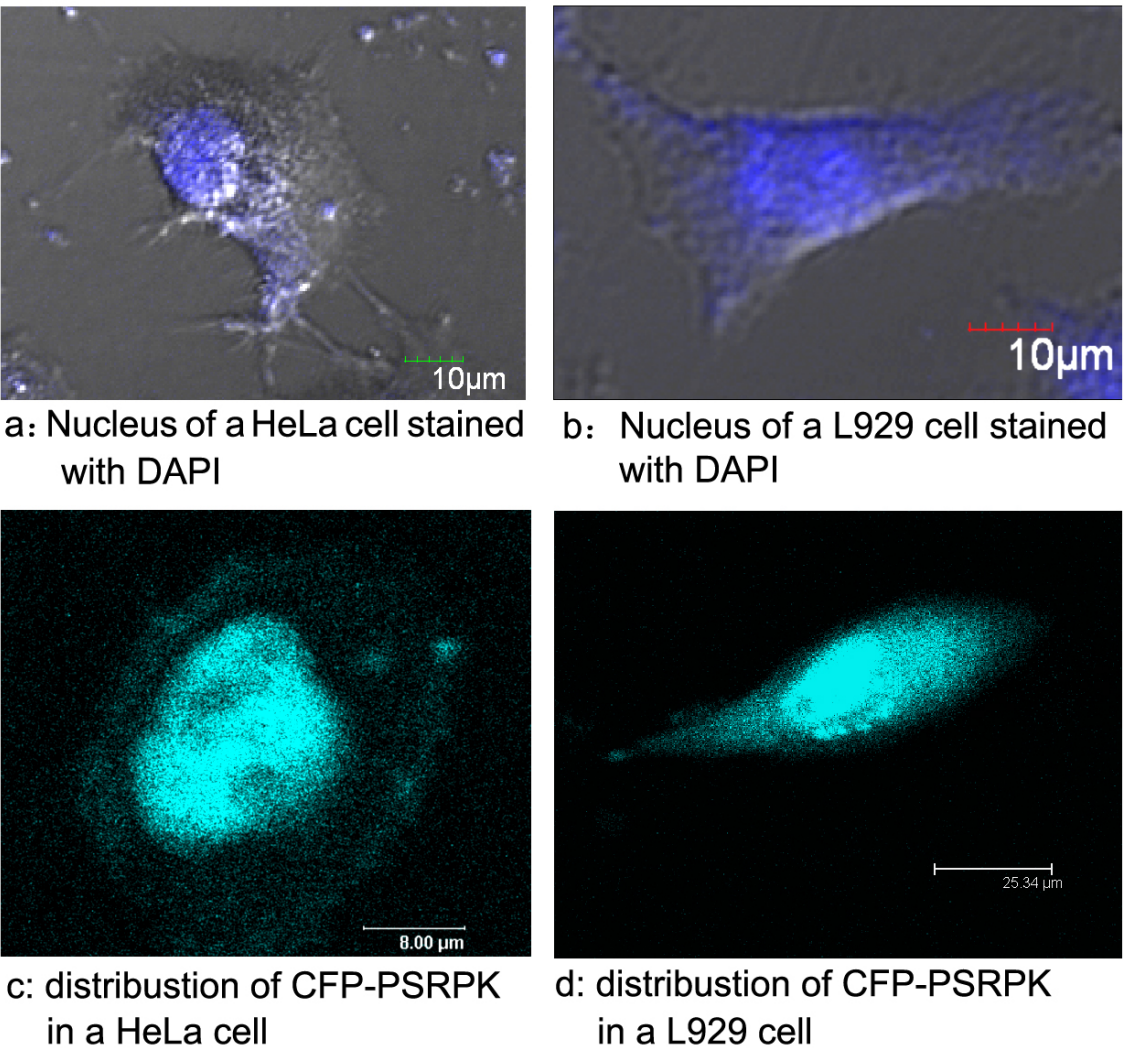
**Schematic diagrams of truncated PSRPKs compared with PSRPK**. **TP1~TP7**: Peptides containing the PSRPK aa 29~426, 1~195, 196~426, 259~426, 197~317, 197~343, and 329~426, respectively; **TP8**: peptides containing 197~317 aa combined with 329~426 aa; **TP9**: peptides containing 1~317 aa combined with 329~426 aa. **N**: acidic divergent motif; 1~28 aa; **CD1**: conserved domain 1; 29~195 aa; **CD2**: conserved domain 2; 259~426 aa; **spacer sequence**: divergent motif between the 2 conserved domains; 196~258 aa. NLS: ^318^PKKGDKYDKTD^328^.

The DAPI-stained nucleus of cells HeLa and L929 transfected with pECFP-*psrpk *were clearly observed under UV excitation (Figure 2a, b). Confocal microscopy showed that the distribution of cyan fluorescent protein (CFP)-PSRPK was non-uniform and that this protein was expressed mainly in the nucleus of the HeLa (Figure 2c) and L929 (Figure 2d) cells.

### Existence of an NLS on the C-terminal conserved domain of PSRPK

**Figure 3:**
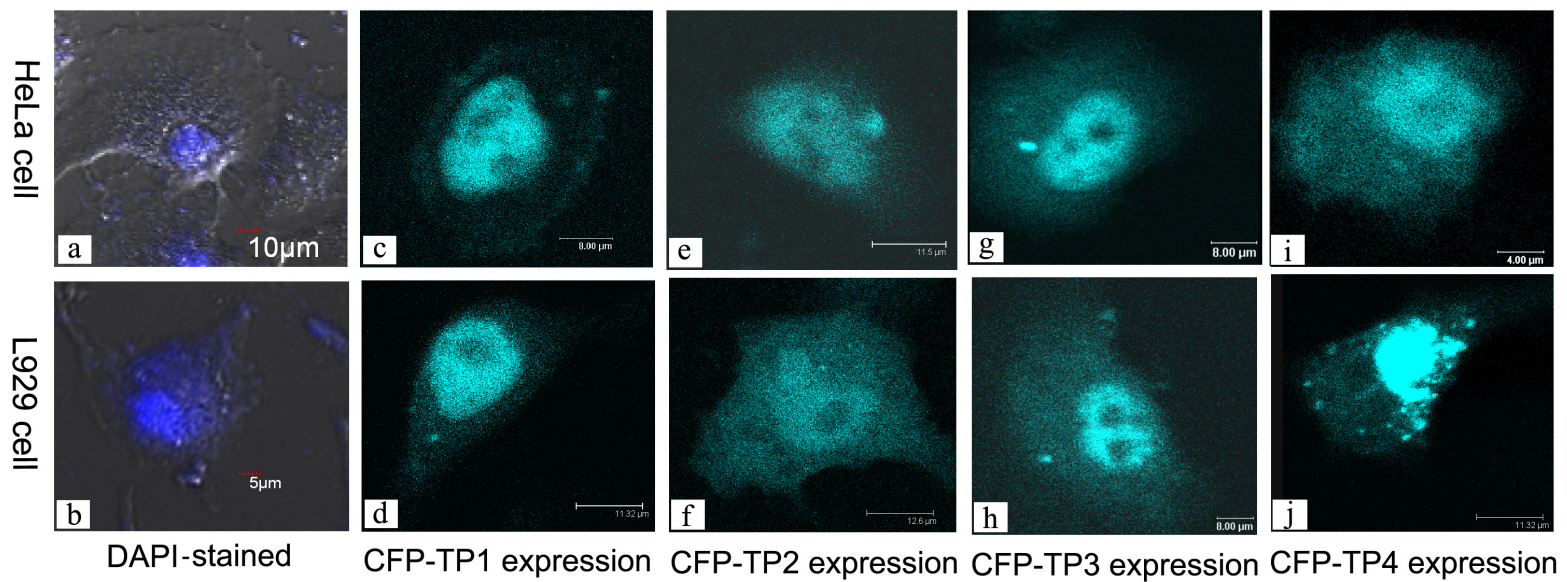
**Distribution of CFP-TPs in the mammalian cells HeLa and L929 observed under a confocal microscope (TCS SP2, Leica)**. The images a and b show DAPI staining nucleus of cells HeLa and L929; the images c and d, e and f, g and h, and i and j show CFP-TP1, CFP-TP2, CFP-TP3, and CFP-TP4 expressions in the HeLa and L929 cells, respectively.

The expression and distribution of CFP fusion TP1, TP2, TP3, and TP4 in mammalian cells were observed by confocal microscopy. Compare with the DAPI staining of the nucleus (Figure 3a, b), the distribution of CFP-TP1 in HeLa (Figure 3c) and L929 (Figure 3d) cells was similar to that of CFP-PSRPK, i.e., both mainly accumulated in the nucleus. This suggested that the deletion of ≥ N had no significant effect on the nuclear accumulation of PSRPK. The distributions of CFP-TP3 and CFP-TP4 in HeLa (Figure 3g, i) and L929 (Figure 3h, j) cells were similar to those of CFP-PRPK and CFP-TP1, i.e., both mainly accumulated in the nucleus. This indicated that PSRPK continued to accumulate in the nucleus despite the deletion of ≥ N, CD1, and the spacer sequence and that CD2 contains an NLS peptide. Unlike the distributions of CFP-PSRPK, CFP-TP1, CFP-TP3, and CFP-TP4, CFP-TP2 in HeLa (Figure 3e) and L929 (Figure 3f) cells exhibited diffuse distribution. The results above indicated that the ≥ N, CD1, and spacer sequence deletions did not influence nuclear localization of PSRPK and that an NLS was located in the C-terminal conserved domain of PSRPK.

### PSRPK NLS was located in the ^318^PKKGDKYDKTD^328 ^sequence

**Figure 4:**
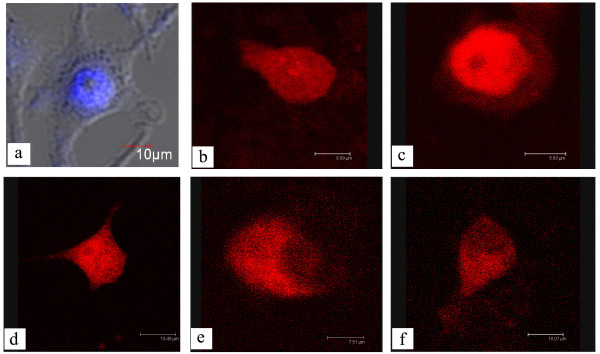
**Distribution of RFP-TP5~RFP-TP9 in L929 cells observed by a confocal microscope (TCS SP2, Leica)**. Only RFP-TP6, which contained ^318^PKKGDKYDKTD^328^, accumulated in the nucleus. a: Nucleus of a pDsRed-*tp6*-transfected L929 cells stained with DAPI and observed under a BX51 fluorescent microscope (Olympus, 400×), while TP8 and TP9 are peptides obtained by the deletion of ^318^PKKGDKYDKTD^328^from TP3 and PSRPK, respectively. b, c, d, e, and f: Expressions of RFP-TP5~RFP-TP9 in L929 cells.

Confocal microscopy revealed that RFP-TP5 and RFP-TP7 were uniformly distributed in the L929 cells (Figure 4b, d), indicating that there was no NLS in the TP5 and TP7 sequences. However, RFP-TP6 mainly accumulated in the nucleus (Figure 4c), indicating that an NLS existed in TP6. By comparing the amino acid sequences of TP5, TP6, and TP7, we primarily confirmed that PSRPK NLS was located in ^318^PKKGDKYDKTD^328^. RFP-TP8 and RFP-TP9 were mainly distributed in the cytoplasm (Figure 4e, f); this further indicated that PSRPK and TP3 lost the ability of nuclear localization after the deletion of the abovementioned sequence. Thus, we could confirm that ^318^PKKGDKYDKTD^328 ^was the NLS sequence of PSRPK.

### The NLS sequence of PSRPK contained a Ω-loop motif

**Figure 5:**
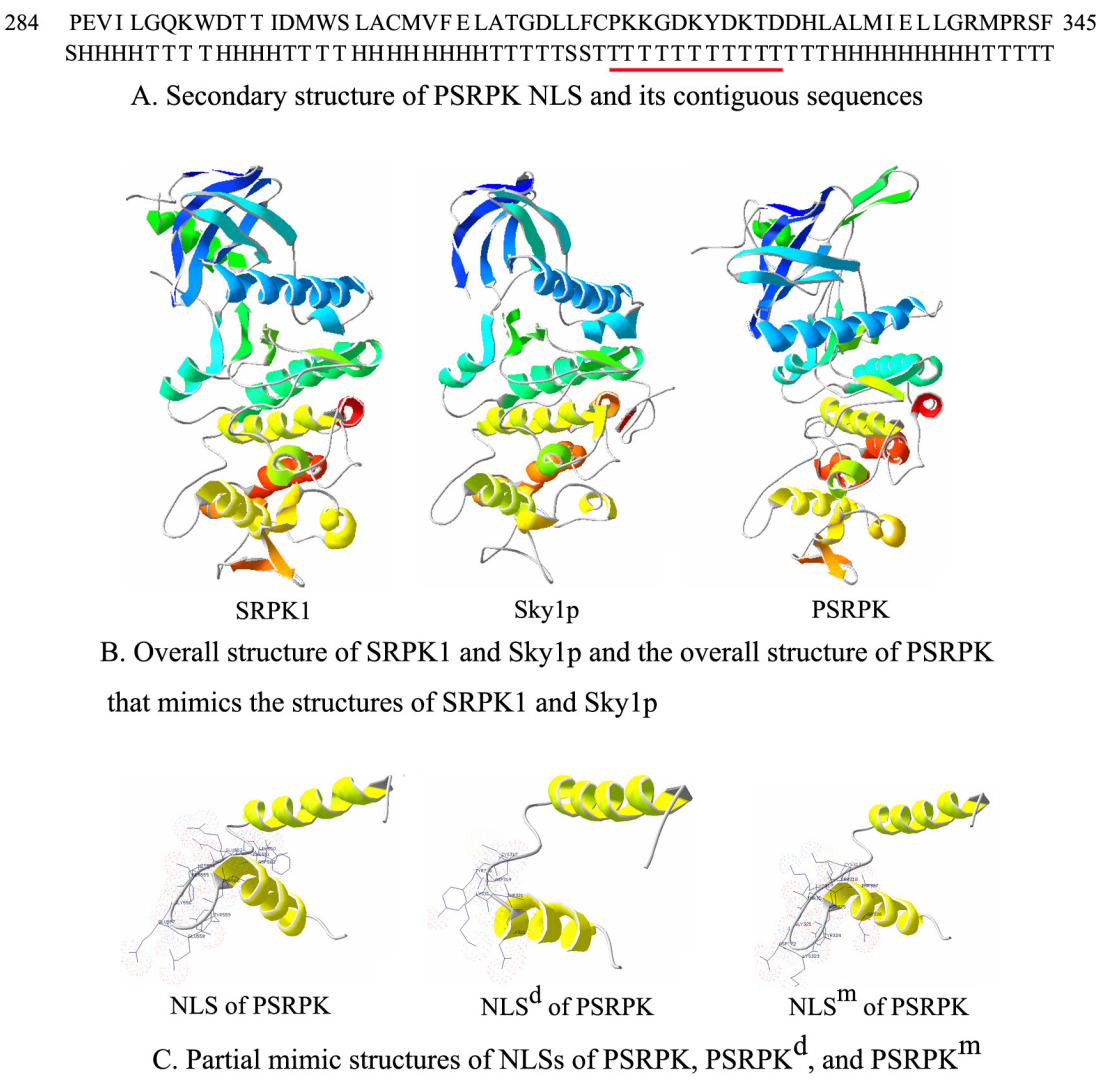
**A: Secondary structure of PSRPK NLS, as predicted by DNASIS v2.5 Demo**. The capital H, S and T blow the sequence represent the corresponding amino acid above has more probability to form helix, sheet or turn. B: The overall structure of PSRPK that mimics the structures of SRPK1 and Sky1p, as predicted by SWISS-MODEL online and displayed by SWISS-Pdb viewer compared with the overall structure of SRPK1 and Sky1p [17-20]. C: **NLS**, the NLS of PSRPK; NLS^d^, the NLS of PSRPK without ^318^PKKGDK^323^; **NLS**^m^, the NLS of PSRPK with the K^320 ^→ T^320 ^mutation.

Analysis of the secondary structure of ^318^PKKGDKYDKTD^328 ^within PSRPK by DNASIS v2.5 Demo revealed that it was a β-turn (Figure 5A). Based on the crystal structure of SRPK1 and Sky1p [17], the tertiary structure of PSRPK (Figure 5B) was predicted using SWISS-MODEL software [18-20]. Figure 5B shows that the structure ^318^PKKGDKYDKTD^328 ^mimics that of the corresponding sequences of SRPK1 and Sky1p. All the 3 sequences are located in the C-terminal loop motif. The spatially near K^320 ^and D^325 ^may form a salt-bridge via their side chains, causing the ^318^PKKGDKYDKTD^328 ^to form a stable Ω-loop. This structure of ^318^PKKGDKYDKTD^328 ^changes after ^318^PKKGDK^323 ^deletion or K^320 ^→ T^320 ^mutation, as predicted by SWISS-MODEL software (Figure 5C). ^318^PKKGDK^323 ^deletion causes damage to the Ω-loop motif, and K^320 ^→ T^320 ^mutation damages the salt-bridge in the Ω-loop.

**Figure 6:**
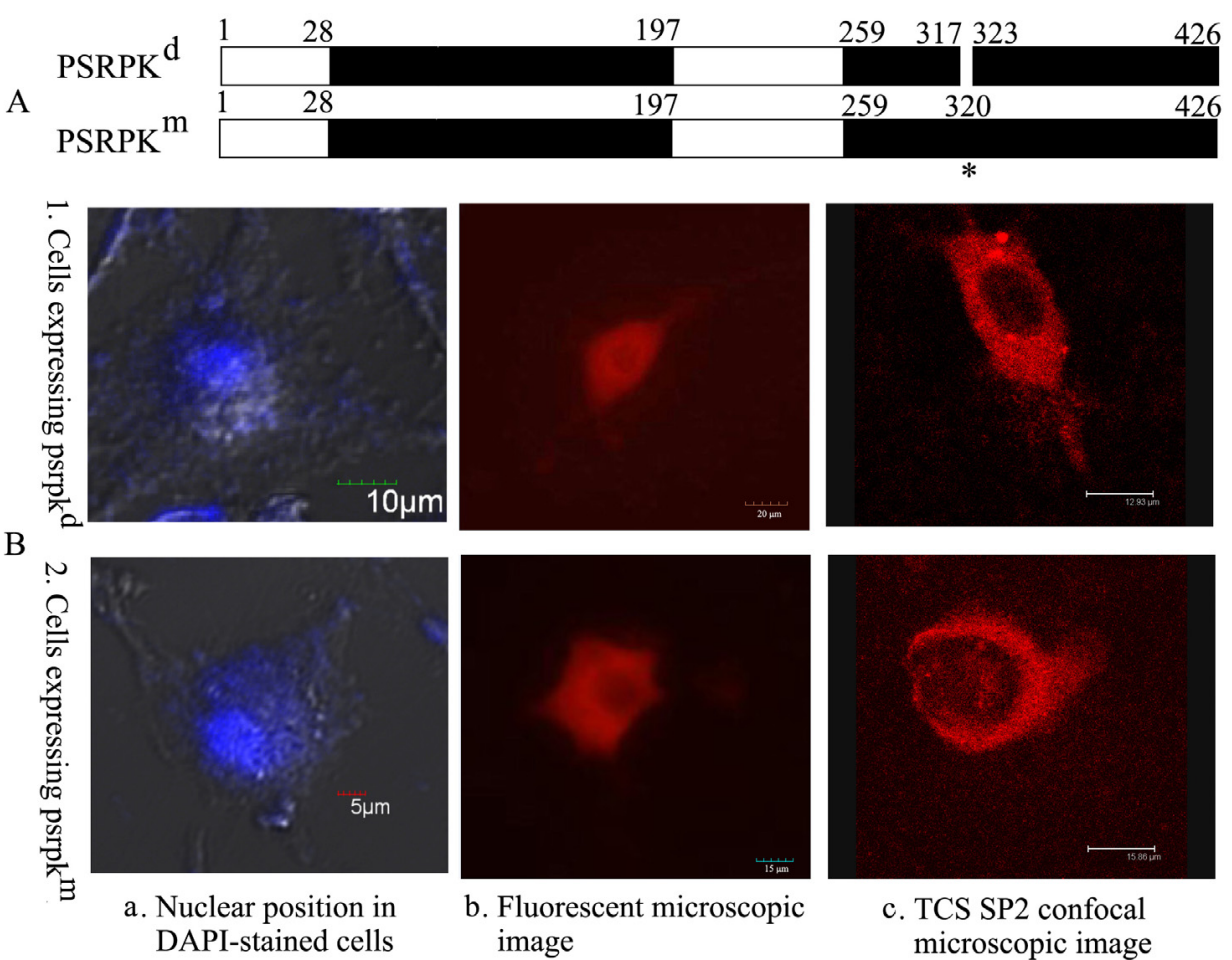
**A: Schematic diagrams of default PSRPK and mutant PSRPK compared with PSRPK**. B: Distribution of RFP-PSRPK^d ^and RFP-PSRPK^m ^expression in L929 cells. PSRPK^d^, default PSRPK without ^318^PKKGDK^323^; PSRPK^m^, mutant PSRPK with the K^320 ^→ T^320^mutation. 1 and 2: Distribution of RFP-PSRPK^d ^and RFP-PSRPK^m ^expressions in L929 cells observed under a confocal microscope (TCS SP2, Leica) compared with their position in the nucleus in DAPI-stained cells observed under a BX51 fluorescent microscope (Olympus, 400×).

The fluorescent fusion proteins PSRPK^d ^and PSRPK^m ^in the L929 cells are shown in Figure 6. Compare with the DAPI staining of the nuclear (Figure 6B1a, B2a), the fluorescent signal of RFP-PSRPK^d ^and RFP-PSRPK^m ^was observed in cytoplasm (Figure 6B1b, B2b). Further, confocal microcopy revealed that RFP-PSRPK^d ^and RFP-PSRPK^m ^were mainly distributed in the cytoplasm (Figure 6B1c, B2c); this indicated that the ^318^PKKGDK^323 ^deletion or K^320 ^→ T^320 ^mutation destroyed the structure of PSRPK NLS, resulting in PSRPK losing its ability of nuclear localization.

## Discussion

### PSRPK has an NLS sequence

Ding et al. [11] studied the cell localization of mammalian SRPKs and found that almost all SRPKs without spacer sequences are accumulated in the nucleus, indicating that there is a cytoplasm localization signal in the spacer sequence between the conserved domains. Kuroyanagi et al. [5] speculated that mouse SRPK1 might have 2 potential NLSs that are located in 11~21 aa and 265~277 aa; thus, mSRPK2 may have a potential NLS in 264~276 aa. However, direct evidence of NLS sequences in SRPK family members has been lacking thus far. Laser scanning confocal microscopy revealed that RFPs of default PSRPKs containing ^318^PKKGDKYDKTD^328 ^mainly accumulated in the nucleus of mammalian cells, while the PSRPKs in which the abovementioned sequence was deleted did not accumulate in the nucleus. This indicated that the NLS of PSRPK was located in the ^318^PKKGDKYDKTD^328 ^sequence of the C-terminal conserved domain.

Further, Ding *et al*. [11] showed that mammalian SRPKs were mainly distributed in the cytoplasm. However, the results obtained in our study showed that PSRPK was mainly expressed in the nucleus of the mammalian cells. A comparison of the primary structure of PSRPK and other SRPKs revealed that the conserved domains were almost identical; however, the nonconserved ≥ N and spacer sequence differed among the kinases. A cytoplasm localization signal was present in the spacer sequences of mammalian SRPKs. Further, the spacer sequence of PSRPK was considerably smaller than that of mammalian SRPKs. This difference might be the major reason why PSRPK cannot anchor itself in the mammalian cytoplasm.

### Function of the PSRPK NLS is related to the loop motif

A classic NLS (cNLS) comprises a monopartite or bipartite signal. A monopartite NLS contains 1 cluster of basic residues, while a bipartite NLS contains 2 clusters of basic residues [21]. Similar to the sequence feature of a monopartite NLS, the NLS of PSRPK is rich in basic residues. A monopartite NLS sequence that is located at the terminal of a protein is usually in a coil, while that located in the interior of a protein is usually in a loop. The PSRPK NLS is also located in the basic loop between 2 α-helixes (Figure 5). The close side chains of K^320 ^and D^325 ^form a salt bridge, thus forming a stable NLS Ω-loop motif (Figure 5C). The experimental results showed that PSRPK lost its nuclear localization ability following the deletion of ^318 ^PKKGDK^323 ^or mutation from K^320 ^to T^320^. The deletion or mutation destroyed the Ω-loop motif of PSRPK, thus suggesting that the loop structure of PSRPK NLS controls the nuclear localization of PSRPK. The ^318^PKKGDKYDKTD^328 ^sequence, which corresponds to the nonconserved sequences of SRPK1 and Sky1p, is located in the loop of an HLHM [18,19] (Figure 5B). Therefore, studies on NLSs of SRPKs should focus on the loops in HLHMs.

## Conclusion

In this study, by truncation of PSRPK protein, deletion of and single amino acid substitution in a putative NLS and transfection of mammalian cells, we demonstrated that the ^318^PKKGDKYDKTD^328 ^peptides localized in the C-terminal conserved domain of PSRPK with the Ω-loop structure could play a crucial role in the NLS function of PSRPK.

## Authors' contributions

SL carried out the design of the study, participated in all studies, and drafted the manuscript. ZZ carried out the most of the tests. ZL carried out confocal microscopy scanning. QO constructed partial plasmids. JZ participated all of the tests. ST participated in the study, and proofreading the manuscript. MX, conceived of the study, participated in its design and coordination.

## References

[B1] Aubol BE, Chakrabarti S, Ngo J, Shaffer J, Nolen B, Fu XD, Ghosh G, Adams JA (2003). Processive phosphorylation of alternative splicing factor/splicing factor 2. Proc Natl Acad Sci USA.

[B2] Gui JF, Lane WS, Fu XD (1994). A serine kinase regulates intracellular localization of splicing factors in the cell cycle. Nature.

[B3] Gui JF, Tronchere H, Chandler SD, Fu XD (1994). Purification and characterization of a kinase specific for the serine- and arginine-rich pre-mRNA splicing factors. Proc Natl Acad Sci USA.

[B4] Wang HY, Lin W, Dyck JA, Yeakley JM, Songyang Z, Cantley LC, Fu XD (1998). SRPK2: a differentially expressed SR protein-specific kinase involved in mediating the interaction and localization of pre-mRNA splicing factors in mammalian cells. J Cell Biol.

[B5] Kuroyanagi N, Onogi H, Wakabayashi T, Hagiwara M (1998). Novel SR-protein-specific kinase, SRPK2, disassembles nuclear speckles. Biochem Biophys Res Commun.

[B6] Tang Z, Yanagida M, Lin RJ (1998). Fission yeast mitotic regulator Dsk1 is an SR protein-specific kinase. J Biol Chem.

[B7] Siebel CW, Feng L, Guthrie C, Fu XD (1999). Conservation in budding yeast of a kinase specific for SR splicing factors. Proc Natl Acad Sci USA.

[B8] Kuroyanagi H, Kimura T, Wada K, Hisamoto N, Matsumoto K, Hagiwara M (2000). SPK-1, a C. elegans SR protein kinase homologue, is essential for embryogenesis and required for germline development. Mech Dev.

[B9] Portal D, Lobo GS, Kadener S, Prasad J, Espinosa JM, Pereira CA, Tang Z, Lin RJ, Manley JL, Kornblihtt AR, Flawiá MM, Torres HN (2003). Trypanosoma cruzi TcSRPK, the first protozoan member of the SRPK family, is biochemically and functionally conserved with metazoan SR protein- specific kinases. Mol Biochem Parasitol.

[B10] de la Fuente van Bentem S, Anrather D, Roitinger E, Djamei A, Hufnagl T, Barta A, Csaszar E, Dohnal I, Lecourieux D, Hirt H (2006). Phosphoproteomics reveals extensive in vivo phosphorylation of Arabidopsis proteins involved in RNA metabolism. Nucleic Acids Res.

[B11] Ding JH, Zhong XY, Hagopian JC, Cruz MM, Ghosh G, Feramisco J, Adams JA, Fu XD (2006). Regulated cellular partitioning of SR protein-specific kinases in mammalian cells. Mol Biol Cell.

[B12] Koizumi J, Okamoto Y, Onogi H, Mayeda A, Krainer AR, Hagiwara M (1999). The subcellular localization of SF2/ASF is regulated by direct interaction with SR protein kinases (SRPKs). J Biol Chem.

[B13] Misteli T, Cáceres JF, Clement JQ, Krainer AR, Wilkinson MF, Spector DL (1998). Serine phosphorylation of SR proteins is required for their recruitment to sites of transcription in vivo. J Cell Biol.

[B14] Yeakley JM, Tronchère H, Olesen J, Dyck JA, Wang HY, Fu XD (1999). Phosphorylation regulates in vivo interaction and molecular targeting of serine/arginine-rich pre-mRNA splicing factors. J Cell Biol.

[B15] Tang Z, Tsurumi A, Alaei S, Wilson C, Chiu C, Oya J, Ngo B (2007). Dsk1p kinase phosphorylates SR proteins and regulates their cellular localization in fission yeast. Biochem J.

[B16] Liu SD, Kang K, Zhang JH, Ouyang QL, Zhou ZL, Tian SL, Xing M (2009). A novel SR protein kinase from *Physarum polycephalum *phosphorylates specificlly on RS domain of human SR protein ASF/SF2. Acta Biochimica et Biophysica Sinica.

[B17] Schwede T, Kopp J, Guex N, Peitsch MC (2003). SWISS-MODEL: an automated protein homology- modeling server. Nucleic Acids Research.

[B18] Lukasiewicz R, Velazquez-Dones A, Huynh N, Hagopian J, Fu XD, Adams J, Ghosh G (2007). Structurally unique yeast and mammalian serine-arginine protein kinases catalyze evolutionarily conserved phosphorylation reactions. J Biol Chem.

[B19] Nolen B, Yun CY, Wong CF, McCammon JA, Fu XD, Ghosh G (2001). The structure of Sky1p reveals a novel mechanism for constitutive activity. Nat Struct Biol.

[B20] Ngo JC, Gullingsrud J, Giang K, Yeh MJ, Fu XD, Adams JA, McCammon JA, Ghosh G (2007). SR protein kinase 1 is resilient to inactivation. Structure.

[B21] Lange A, Mills RE, Lange CJ, Stewart M, Devine SE, Corbett AH (2007). Classical nuclear localization signals: definition, function, and interaction with importin alpha. J Biol Chem.

